# Trend of Medical Tourism Publications: An Attempt to Explore the Involved Academic Disciplines and Interests

**Published:** 2018-02

**Authors:** Ladan ROKNI, Sam-Hun PARK

**Affiliations:** Asia Contents Institute, Konkuk University, Seoul, South Korea

**Keywords:** Medical tourism, Health travel, Publication trend, Academic categories, ISCED

## Abstract

**Background:**

Medical tourism suffers from the lack of a consensus regarding the involved categories. This study aimed to address this gap from the academic disciplines and publications perspective.

**Methods:**

Totally 1954 citations were identified through a formula of keyword search of SCOPUS. In order to classify the various subject areas, we followed the international standard classification of education (ISCED) developed by UNESCO. Moreover, the trends of publications were identified based on their popularity between 2000 and 2017.

**Results:**

The category with the most interests on publication about medical tourism was ‘health and welfare’, followed by ‘social science’. Even though various disciplines were involved in the medical tourism, it seems that a downward trend has been experienced since 2015.

**Conclusion:**

The identified key trends of medical tourism publications will benefit researchers exploring the categories of medical tourism or health travel. The results contribute to advance the state of knowledge from the academic perspective.

## Introduction

Medical tourism emerged as a new type of healthcare mobility (1, 2) and several definitions have been presented by researchers, yet there is a dearth of evidence on a widely accepted definition or even an accepted title. This shortage might be due to the nature and variation of the main objectives of this mobility. It involves people who are neither tourists nor patients (3) (claimed by the website of medical travel quality alliance) and they travel of the borders with the aim of medical treatment (4), likewise, a definition of ‘medical tourism’ refers to traveling across international borders to access private medical care (5). Meanwhile, it is not clear as to whether such definitions involves health tourism and wellness tourism as well, or these concepts should be regarded as different, and where are the boundaries.

This barrier not only does refer to the definition, but also the associated industry and service sector are suffering to clarify the involved categories. Those companies active in medical tourism are ‘integrate into the wider tourism industry’ (2). Many countries are trying to promote a ‘global marketplace for health services’(6), but they might face ‘stagnancy’ after a period of ‘prosperity’ and one determinant could, possibly, be the negative impact of unfamiliarity with the procedure. In fact, there is not an accurate procedure and it is not clear as to whether medical tourism is a new trend in medical service (7), tourism (8), industry, business or service sector. Referring to the on-going scholarly debates, also, a critique has emerged that categories of medical tourism are not identified and defined well (9). Therefore, it seems that medical tourism should be regarded as a multidisciplinary program; however, there is lack of evidence on those categories involved.

In order to address this shortage, a constructive attempt would be to explore the academic disciplines and program that have been included in the publications of medical tourism and health travel. It would also imply on the background of those researchers that are interested in medical tourism and accordingly would lead to the backbone of this multidisciplinary service from the academician and publication perspective; moreover, it could potentially result in a more accurate definition of medical tourism which is based on the practical studies and the available factual information.

In order to classify the comprehensive research of medical tourism, a well-established category was required. From the perspective of journalism and academic publications, there are various types of classification. The most widely accepted classification is the international standard classification of education (ISCED) a framework developed by United Nations International Family of Economic and Social (UNESCO) (10). This classification is the reference for organizing education programs.

Accordingly, this study aimed to investigate the backbone of academic publications on medical tourism in order to clarify the variation of the involved disciplines. The objectives were 1) to determine the disciplines of the most renowned publications, 2) to determine the boundaries and quantity of those categories that were interested in medical tourism publications, 3) to explore the popularity of medical tourism publications during a period of time, and finally, 4) to clarify the trend of medical tourism scholarly publications.

## Methods

This exploratory research was conducted focusing on several questions. A comprehensive search was accomplished on the secondary data (publications indexed in SCOPUS) to retrieve the relevant articles. Afterward, data were classified based on the ISCED category. The process of the data collection and analysis are explained in the following sections.

### Data sources and search strategy

We searched the most renowned and well established academic databases. The search strategy was designed based on each database in order to maximize sensitivity. The main keywords included: (”Medical Tourism “OR” Health Tourism “OR” Medical Travel”). Searching these keywords in Google scholar, 16,800 results were found, also SCOPUS and science direct showed 1,954 and 612 results respectively. The information was updated two months before the publication so that the latest data could be analyzed.

Given that this study was not searching for the contents of publications, rather it aimed to find the quantity and the main trend of this international health service and international patient’s mobility, we only focused on the data indexed in SCOPUS. The reason to focus only on one database was to cover the main trend among the mostly referable publications, also to exclude the possibility of article duplication in several databases. Moreover, the classifications of subjects differ based on each database regulations. Hence we decided to focus on one database and to classify the data in the ISCED category. On the other hand, finding the research trend through meta-analysis was not possible due to the heterogeneity of the topic (11).

The references used in this study were all published during a time period of 2000 and 2017. Thus, this does not imply that those publications before 2000 were not significant, rather in the initial search we found that the main publication in this arena has been mainly started since 2005.

### Data analysis

As noted earlier, all data were collected from the database of SCOPUS. Searching the abovementioned formula, 1954 documents were found. These articles were analyzed based on two perspectives of ‘academic discipline’ and year of publication.

The SCOPUS classify the documents from the perspective of ‘subject area’ which is designed based on the indexed keywords of each publication. Totally 27 ‘subject areas’ were presented for that formula of keywords we searched. In order to find the trend in an internationally accepted category, we attempted to reclassify those 27 categories (subject areas) based on the ISCED. This category which is developed by UNESCO seems to be the most comprehensive well-established framework for ‘analyzing cross-nationally comparable statistics on education’ (10). We applied the 2013 version, which mainly focused on the fields of education. Totally 10 main categories were introduced by ISCED; hence 27 subject area of SCOPUS have been classified accordingly (Table 1). Furthermore, searching the date of publications, we aimed to clarify the popularity of a specific trend during a period of time. Therefore, the quantity of each category presents the introduction or popularity of a trend to medical tourism arena.

**Table 1:** Classifying the subject area of medical tourism based on their associated education category

***ISCED classification***	***Health & Welfare***	***Social Science, Journalism & Information***	***Business, Administration & Law***	***Natural Sciences, Mathematic & Statistics***	***Art & Humanities***	***Engineering, Manufacturing & Construction***	***Information & Communication Technologies***	***Agriculture, Forestry, Fisheries & Veterinary***
Subject Area based on SCOPUS	Medicine (1230)	Social Science (503)	Business, Management, Accounting (307)	Biochemistry, Genetic & Molecular Biology (100)	Art & Humanities (119)	Engineering (69)	Computer science (57)	Agricultural and Biological Sciences (16)
	Nursing (143)	Economic, Econometric & finance (120)		Environmental Science (56)		Chemical Engineering (11)		Veterinary (2)
	Health Professions (49)	Decision Sciences (20)		Earth & Planetary Science (47)		Materials Science (11)		
	Immunology and Microbiology (18)	Psychology (14)		Pharmacology, Toxicology and Pharmaceutics (34)		Energy (7)		
	Dentistry (15)			Mathematics (14)				
	Neuroscience (6)			Physics (2)				
Total	**1461**	**657**	**307**	**253**	**119**	**98**	**57**	**18**
Multidisciplinary	20							
Undefined	2							
Note	Education and Services subjects had zero rate							

## Results

Totally, 1954 citations were searched; all were published between 2000 and 2017 and indexed in SCOPUS. The quantities of publications and the classification are listed in Table 1. It represents the distribution of the searched publications based on their subject area and ISCED framework.

The category of ‘health and welfare’ has the highest amount of publications by 1461 cited articles. It was followed by the category of ‘social science’ with 657 publications. Third in terms of publication were those related to ‘business, administration and law’ counted for 307 publications; afterward, the category of ‘natural sciences and statistics’ with 253 publications and ‘art and humanities’ with 119, entitled as the fourth and the fifth most involved categories. The three categories with the fewest interests in publications, meanwhile, were ‘engineering and construction’, ‘information technologies’ and ‘agriculture’ with the rates of 98, 57 and 18, respectively.

The trends of publications have been started since 2005 by the researchers with interest in the category of health and welfare; however, there are two critical periods of time for the other categories. It seems that 2008 and 2009 were the starting points for the researchers coming from different other disciplines to conduct their research regarding medical tourism. The second critical time period was 2015, the peak time for medical tourism publications among several categories, though those interests were followed by sharp downward trends. This pattern is true for almost all categories.

**Fig. 1:**
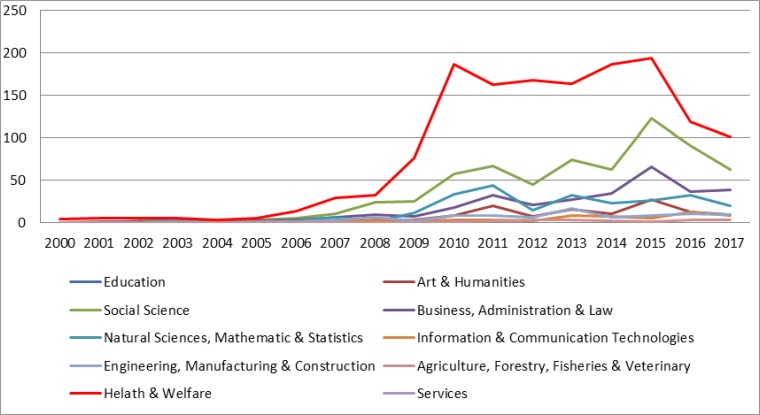
The distribution of medical tourism publications between 2000 and 2017, classified based on the ISCED developed by UNESCO

Following the trends of all 10 ISCED (presented in Fig. 1), it was clear that ‘health and welfare’ category has been by far the most involved field of publication in terms of medical tourism. From a negligible 5 in 2005, the number of publication claimed to 32 in 2008, before soaring to just less than 200 in 2010. It was followed by a period of fluctuations and then a sharp decrease since 2015.

Second ISCED field in terms of publication was social science which experienced an upward trend before 2015, though; there were several fluctuations during the whole period. The general trend of this category was similar to that of natural science. Their trends were comparable during these years, though, with lower publication rates for the latter one. It continued until 2014, however, the gap between them widened after this time as publications in social science faced an exponential growth before sliding down to 63, while that of natural science remained approximately constant.

The researchers with the background of business and administration started their interests to the medical tourism publications with a lower rate of natural science; nevertheless, the former category has exceeded that of the latter one. This trend continued until the category of business reached a peak of 66 publications in 2015. Though, in the following year number of publications decreased to half of that and was just similar to natural science. Other categories were art/humanities, engineering and information tech. with the publications’ peaks in 2015, 2013 and 2016, respectively.

Two categories of ‘education’ and ‘service’ have no publication during this period. However, both seem to be critical for medical tourism. It should be taken into account that having not a single publication does not imply that these two categories are not significant. According to the search we conducted, mainly on the publications with these two themes, they were mostly categorized as social science or business and management. It is important to notice that we followed the classification developed by SCOPUS rather than analyzing the publications one by one. Therefore, the category presented in Table 1 might differ based on the other classifications. Moreover, 20 articles were categorized as ‘multidisciplinary’.

## Discussion

This study aimed to explore those academic categories that have been involved in the publications of medical tourism/health travel in order to provide a reflection on the medical tourism trend. It seems essential to clarify the academic background of medical tourism and to get familiar with the various aspects of that since there was a call to examine the risks of ‘underrepresenting’ certain voices in the process of providing medical service to patients traveling of borders (9). This attempt could potentially help to address the lack of clarification on the different categories from the perspective of the academic publication.

The result revealed that the main trend of medical tourism publications has been started in the fields of healthcare and medicine. This category, by far, made the main publications of medical and health travel, nevertheless, it failed to remain as potent as earlier time after 2015. Likewise, this year seems to be a critical year for medical tourism publications with the exponential decrease in several categories; the only exception is the category of ‘business, administration and law’. It might imply on the fact that the administration associated domain still suffers from the shortage of clear regulations.

Moreover, this study found that not only medicine and traveling service domains are engaged in medical tourism publications, but also it is interested in various fields of study. Being a multidisciplinary domain of research also implies on its complexity in practice. It has been broadly suggested to consider various key players (12); even there is complexity in a scale of a local company, marketing medical travel (6). There are various requirements, infrastructures, training, and services in this process and only considering ‘the number of medical travelers’ as the key indicator of a destination would mislead the authorities (12). It has been, also claimed that medical tourism is not a ‘unified phenomenon’ (13). Moreover there are several barriers to this international business (14, 15).

Accordingly, it is highly unlikely to attribute the process of medical/health travel to one or two specific domain(s) of research. We could infer from these figures that researchers from various disciplines are interested to conduct their research in the subject of medical tourism.

It should be noted that the associated publications in SCOPUS could not cover all the available information, though; the results seem to be beneficial enough for being generalized and helpful to address a small part of available complexity and ambiguities. Furthermore, since we followed the classification of SCOPUS and ISCED, the results would be comparable with the further studies on other databases and other classifications.

## Conclusion

The wide ranges of the involved academic category in medical tourism publications were classified and the trends of those interests were presented. It could also imply on the academic backbone of this arena.

## Ethical considerations

Ethical issues (Including plagiarism, informed consent, misconduct, data fabrication and/or falsification, double publication and/or submission, redundancy, etc.) have been completely observed by the authors.
